# A retrospective cohort study on oesophageal food bolus obstruction in the North Denmark region in 2021—two thirds were never diagnosed with a cause

**DOI:** 10.1186/s12876-023-03077-8

**Published:** 2024-01-02

**Authors:** Jacob Holmen Terkelsen, Martin Hollænder, Kasper Bredal, Sara Munk Nielsen, Kristoffer Vittrup Koed Thomsen, Amanda Baggerman, Emilia Ofverlind, Alptug Mertcan Koc, Hannah Pakes, Marco Bassam Mahdi, Sanne Ørnfeldt Larsen, Vanessa Parra Gonzalez, Johannes Riis, Line Tegtmeier Frandsen, Dorte Melgaard, Anne Lund Krarup

**Affiliations:** 1 Department of Emergency Medicine and Trauma Center, Aalborg University Hospital, Hobrovej 18-22, DK-9000 Aalborg, Denmark; 2 School of Medicine and Health, Aalborg University, Aalborg, Denmark; 3 Department of Gastroenterology, North Denmark Regional Hospital, Hjørring, Denmark; 4 Department of Gastroenterology and Hepatology, Aalborg University Hospital, Aalborg, Denmark; 5 Faculty of Clinical Medicine, Aalborg University, Aalborg, Denmark

**Keywords:** Food Bolus obstruction, Food Bolus Impaction, Oesophagus, Upper Endoscopy, Eosinophilic oesophagitis, Oesophageal cancer

## Abstract

**Background:**

Food bolus obstruction (FBO) leading to hospital treatment is often associated with eosinophilic oesophagitis (EoE), stenosis, or oesophageal cancer (1). Danish national guidelines recommend that patients with FBO undergo a diagnostic upper endoscopy within two weeks of presentation to exclude possible malignancy, and histological evaluation of eight biopsies (2, 3).

**Aims:**

The aims of this study were to (1) report the incidence and describe the causes and treatment of FBO in the North Denmark Region (NDR), (2) determine the proportion of patients who underwent upper endoscopy and biopsy according to regional and national guidelines, and (3) identify International Classification of Diseases 10th Revision (ICD-10) diagnosis and procedure codes applied to the hospital visits due to FBO in the NDR.

**Methods:**

Among all acute hospital visits in the NDR in 2021, all visits with ICD-10 codes possibly reflecting FBO, as well as a random sample of 14,400 visits with unspecific ICD-10 codes (R and Z codes), were screened manually for possible FBO. Diagnosis, follow-up, and treatment of all patients with FBO were recorded.

**Results:**

The median patient age was 66.0 (Q1-Q3: 49.8–81.0) years, and half of the patients had experienced FBO before. Two thirds of patients (66.0%) were never diagnosed with a cause of FBO, followed by 17.3% with EoE. 30% of patients did not undergo upper endoscopy within two weeks of the hospital visit, and 50.7% were never biopsied in the oesophagus. Of 1886 hospital visits with registry ICD-10 codes that possibly reflected FBO, 8.4% were due to FBO, while FBO was present in 0.028% of the random sample of unspecific ICD-10 codes.

**Conclusions:**

Most hospitalized FBO patients in the NDR in 2021 were never diagnosed with a cause. In these patients there is a high risk of overlooked EoE or upper gastrointestinal cancers. The area needs immediate focus and changed routines to improve treatment and prevent new FBO.

## Introduction

Oesophageal obstruction is an indication for emergency upper endoscopy and can be caused by either foreign bodies or food [4]. Oesophageal obstruction due to foreign bodies is not necessarily pathological, since they are not meant to be swallowed. In contrast, oesophageal food bolus obstruction (FBO) is pathological, and is the focus of this study. Clinical manifestations of FBO are acute and may include odynophagia, diffuse chest pain or pressure, choking, vomiting and an inability to swallow liquid or saliva which can lead to drooling. Additionally, the patient may experience respiratory manifestations due to aspiration [5]. FBO and its associated clinical manifestations may pass spontaneously, or be treated conservatively (carbonated drinks), pharmacologically (muscle relaxant injection) and/or by therapeutic endoscopic removal [6]. After resolving acute FBO, the latest international guidelines published in 2016 [1] recommend a diagnostic upper endoscopy including histological evaluation, since underlying oesophageal pathology is found in more than 75% of FBO cases [1]. The most common findings are peptic strictures (> 50%) and eosinophilic oesophagitis (EoE) (≈ 40%), while cancer and motility disorders (e.g., achalasia) are less common [1]. In Denmark, national guidelines recommend upper endoscopy within two weeks after FBO to exclude cancer, with histological evaluation of eight biopsies (on the suspicion of EoE), even with no macroscopical oesophageal pathology [2, 3]. International studies that do not exclude FBO patients who did not require endoscopy [7–11] report lower endoscopy (65.2–86.2%) and/or biopsy rates (16.1–42.2%) than recommended in the latest internationally published guidelines [1]. As these studies included patients before the new guidelines were published it is unknown if the diagnostic evaluation of FBO patients has changed for the better. Additionally, through the daily clinical work in the North Denmark Region and from a previous study reporting insufficient oesophageal histological evaluation in Denmark [12], concerns are raised that FBO is not handled correctly and according to guidelines in Denmark, as no studies on FBO patients in Denmark exist.

The aims of this study were to (1) report the incidence of FBO and describe causes and treatment of FBO in the North Denmark Region (NDR), (2) determine the proportion of patients who underwent upper endoscopy and biopsy according to regional and national guidelines, and (3) identify International Classification of Diseases 10th Revision (ICD-10) diagnosis and procedure codes applied to the hospital visits due to FBO in the NDR.

## Methods

### Study design

This is a retrospective cohort study in which all patients who suffered from FBO in NDR in 2021 were sought identified, and their medical history prior to and following the incident captured.

### Study population

All acute hospital visits from all three emergency departments in the entire NDR 2021 were identified using registries. All selected ICD-10 diagnosis and procedure codes applied at referral or discharge that could have been used for a patient with FBO were included for screening of FBO episodes using manual medical record evaluation (see Table 1). These were categorized as either specific (codes covering hospital visits possibly due to FBO) or unspecific codes (where FBO could not be excluded). Hospital visits with DG codes (neurological disorders and muscular diseases) and no specific codes applied were excluded prior to screening for FBO, as well as visits with codes for enteroscopy, colonoscopy and sigmoidoscopy but not a code for upper endoscopy applied. Inclusion criteria were 1) FBO in the oesophagus including pills, while exclusion criteria were (1) FBO in the pharynx/larynx, (2) foreign bodies in the oesophagus of non-food origin (apart from pills), and (3) age below 18 years. All medical records from hospital visits with specific codes were reviewed in detail, and those who suffered from FBO (based on relevant clinical manifestations and/or upper endoscopy findings) were included in the study population. A sample of 14,400 from the unspecific group of 56,260 hospital visits were also reviewed to find patients with FBO through manual medical record review.

**Table 1:** The use of ICD-10 codes and procedure codes to find patients with food bolus obstruction

ICD-10 codes	Description	Specific codes (possible FBO) (S), unspecific codes (FBO could not be excluded) (U), and excluded codes (E)
DC15-16 + DC26	Neoplasm in the gastrointestinal canal, primarily in the oral cavity and the oesophagus	S
DF06	Organic psychiatric disorders	S
DG00-DG26 + DG30-DG32 + DG35-DG37 + DG51 + DG70-DG73 + DG80-DG83	Neurological disorders and muscular diseases	E
DK20-23	Oesophageal diseases	S
DR	Symptoms and abnormal findings	(U)
- DR139	Dysphagia without any further clarification	S
DT180-181 + DT189	Foreign body/food bolus impaction in the gastrointestinal canal, primarily the oral cavity and the oesophagus.	S
DZ	Factors that influence health and contact with the healthcare system	U
Procedure codes		
KGE	Mediastinal surgery	S
KJC	Oesophageal surgery	S
KJN	Reconstructive surgery following gastrointestinal surgery	S
KJW	Reoperation after gastrointestinal surgery	S
KUJ	Endoscopy of the gastrointestinal canal	S

Abbreviation: FBO; food bolus obstruction, ICD-10; International Classification of Diseases 10th Revision

### Data collection

All medical records of patients with FBO were reviewed in detail, and data was entered into a REDCap database [13, 14] for subsequent data analysis. For upper endoscopy, biopsy and reoccurrence rates, as well as later diagnosis (defined as diagnosis of oesophageal disease/findings after the acute hospital visit), medical records were reviewed with a minimum of 12 months follow-up.

### Data analysis and results

Data analysis was performed on patients with FBO. Normal distributed data were described as mean (standard deviation (SD)) and non-parametric data as median (inter quartile range (IQR)). Analysis was performed in R (R Foundation for Statistical Computing, Vienna, Austria, 2022).

## Results

### FBO patients were often older and had often experienced FBO previously

Table 2 shows the descriptive results of the medical record review of the patients included with FBO. A total of 150 patients met the inclusion criteria. Among these 150 there were 162 episodes of FBO. With 478,262 adult inhabitants in NDR in 2021 the annual incidence of FBO episodes was 33.9/100.000 adults. The median age was 66.0 (Q1-Q3: 49.8–81.0) years and patients were predominantly male (58.7%). More than half of patients (55.3%) had experienced FBO prior to inclusion. First physician to examine the patients with FBO were from departments of Emergency Medicine, Ear Nose and Throat, or Surgery.

**Table 2:** Demographics and examination characteristics of patients with a hospital visit due to FBO in the North Denmark Region in 2021

Patients, n	150
Age at hospital visit, median (Q1-Q3)	66.0 (49.8–81.0)
Male sex, % (n)	58.7% (88)
Previous FBO, % (n)	55.3% (83)
Diagnoses with relevance to FBO prior to hospital visit, % (n)	
No diagnoses	83.3% (125)
Oesophageal stricture	7.3% (11)
Eosinophilic oesophagitis	5.3% (8)
Achalasia	2.0% (3)
Oesophageal or gastric cardia cancer	2.0% (3)
Previous fundoplication surgery	1.3% (2)
Hospitalised during the acute hospital visit, % (n)	70.0% (105)
Patient was initially examined by a physician from, % (n)	
Department of Emergency medicine	56.0% (84)
Department of Ear nose and throat	28.0% (42)
Department of Surgery	13.3% (20)
Nurse	2.0% (3)
Department of Oncology	0.7% (1)
Patient underwent upper endoscopy, % (n)	79.3% (119)
Within the acute hospital visit	62.0% (93)
From discharge to 2 weeks after discharge	6.0% (9)
From 2 weeks to 6 months after discharge	10.0% (15)
Later than 6 months after discharge	1.3% (2)
No upper endoscopy	20.7% (31)
Patient was biopsied in the oesophagus, % (n)	48.7% (73)
Within the acute hospital visit	24.0% (36)
From discharge to 2 weeks after discharge	4.0% (6)
From 2 weeks to 6 months after discharge	17.3% (26)
Later than 6 months after discharge	3.3% (5)
No biopsies taken	51.3% (77)
Number of oesophageal biopsies sampled, median (Q1-Q3)	8.0 (6.0–8.0)
Reoccurrence of oesophageal FBO, % (n)	16.7% (25)

Abbreviations: N; number, Q1; 1st quartile, Q3; 3rd quartile, FBO; food bolus obstruction

### National guidelines on endoscopy and oesophageal biopsies were often not followed

**Fig. 1:**
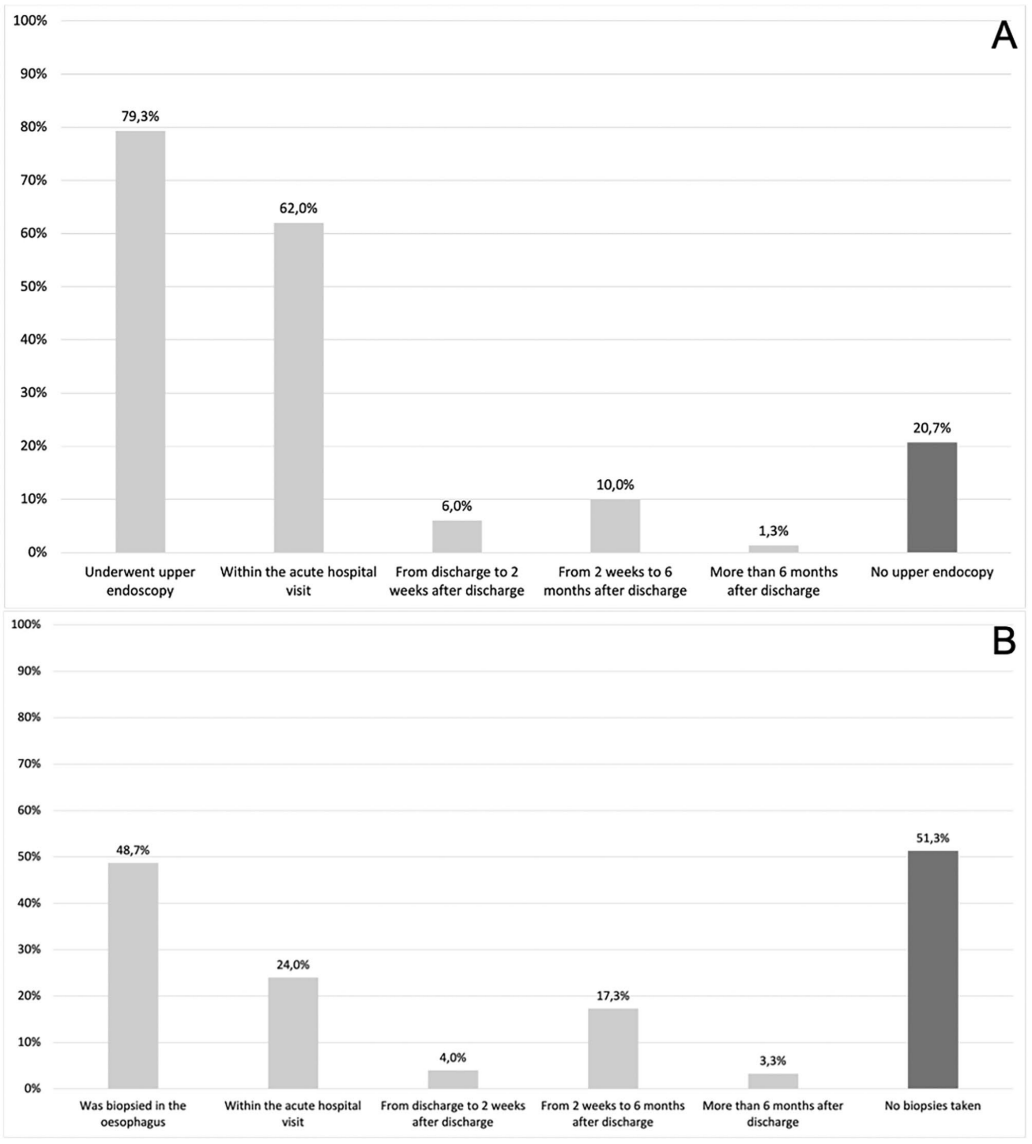
**A + B**: Endoscopy (**A**) and biopsy rates (**B**) among patients presenting to a hospital with FBO in the NDR in 2021. Abbreviations: FBO; food bolus obstruction, NDR; North Denmark region

In total, 32.0% of patients did not undergo upper endoscopy within two weeks of the hospital visit, and 51.3% were never biopsied in the oesophagus (Fig. 1A and B). One sixth experienced FBO reoccurrence within the study period (see Table 2).

### Effective treatment of FBO only involved endoscopic removal in half of patients

Table 3 presents the treatment and the macroscopic findings when upper endoscopies were performed of patients with FBO. Most food boluses consisted of meat or poultry. Nearly 70% were attempted treated with sparkling water, 29.3% with injection of muscle relaxant, and 48.7% underwent endoscopic removal of FBO.

**Table 3:** Endoscopic findings at initial endoscopy and treatment of FBO

Endoscopic findings at initial endoscopy during or after FBO	Within the acute hospital visit, % (n = 93)	From discharge to 2 weeks after discharge, % (n = 9)	Later than 2 weeks after, % (n = 17)
Food bolus	84.9% (79)	22.2% (2)	5.9% (1)
Stricture	12.9% (12)	11.1% (1)	5.9% (1)
Other types of oesophagitis	9.7% (9)	11.1% (1)	5.9% (1)
Edema	9.7% (9)	0%	5.9% (1)
White spots	7.5% (7)	0%	0%
Reflux oesophagitis	6.5% (6)	11.1% (1)	11.8% (2)
Malignancy	2.2% (2)	0%	0%
Schatzki rings	1.1% (1)	22.2% (2)	0%
Exudates	1.1% (1)	0%	0%
Furrows	0%	0%	5.9% (1)
Food causing FBO, % (n)			
Meat (beef, veal or pork)			52.0% (78)
Poultry			13.3% (20)
No clarification or unclear			10.7% (16)
Combinations of food (e.g., meat and potatoes, chicken and rice)			8.7% (13)
Vegetables or fruit			6.0% (9)
Bread			2.7% (4)
Other: tablets, fish or shellfish, candy, or oats			6.7% (10)
Treatment of FBO during the acute hospital visit, % (n)			
Spontaneous resolution			7.3% (11)
Treated with 1st step: sparkling water			68.0% (102)
Sparkling water alone resolved the FBO			25.3% (38)
Treated with 2nd step: injection of muscle relaxant			29.3% (44)
Injection of muscle relaxant alone			0%
Injection of muscle relaxant and sparkling water alone			10.0% (15)
Needed 3rd step: endoscopic removal of FBO			48.7% (73)
Endoscopic removal of FBO alone			14.0% (21)
Oesophageal stent			0.7% (1)
Treatment initiated at discharge, % (n)			
PPI			49.3% (74)
None			51.3% (77)
Antimycotics or antibiotics			6.0% (9)
Oesophageal dilation			2.0% (3)

N; number, FBO; food bolus obstruction, PPI; proton pump inhibitor

### A majority of patients with FBO never got a causal diagnosis

**Fig. 2:**
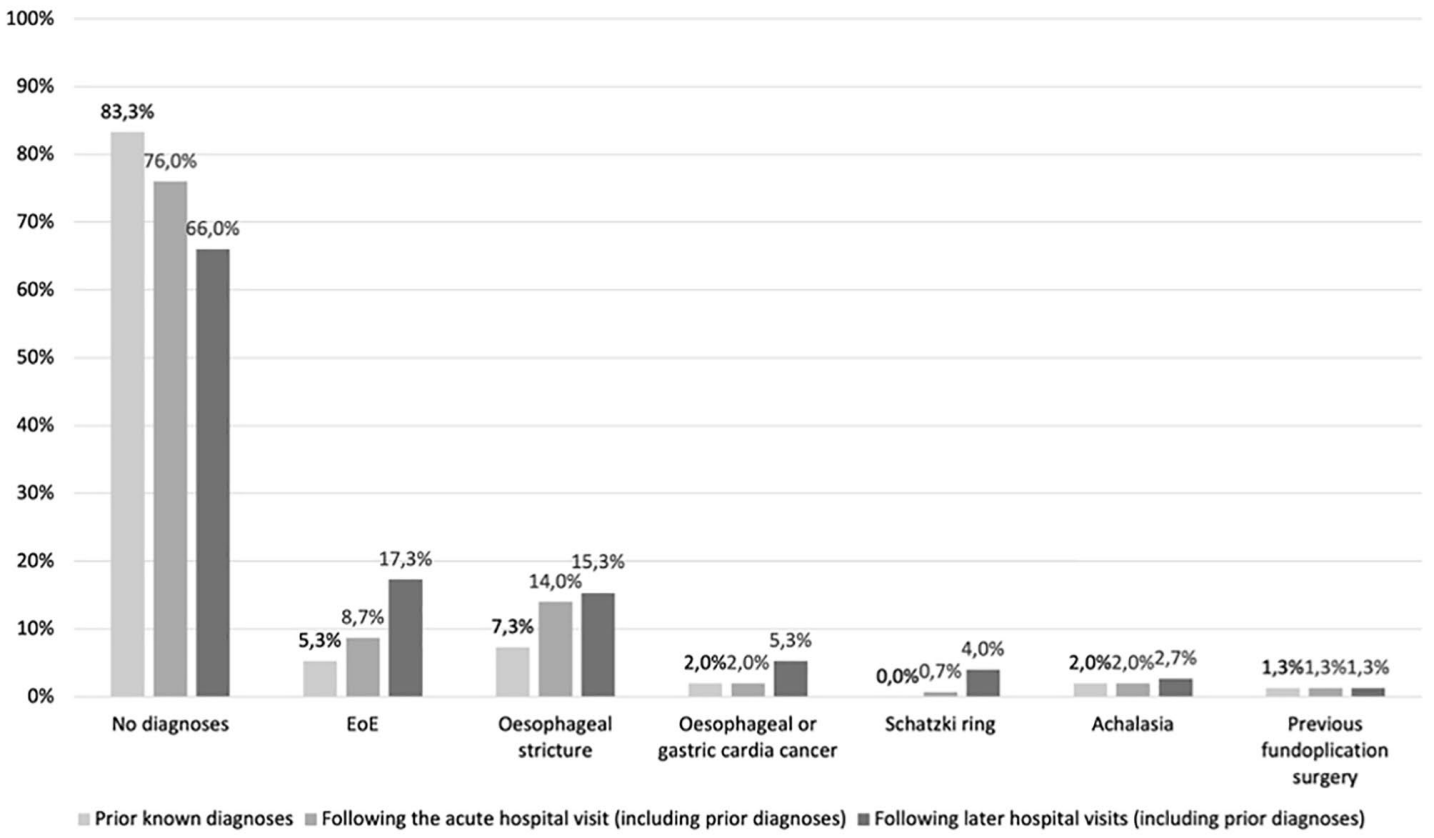
Diagnoses that can cause FBO among patients presenting to a hospital with FBO in the NDR in 2021. Abbreviations: FBO; food bolus obstruction, NDR; North Denmark Region

Table 4 shows the pooled diagnoses as causes of FBO applied either during the hospital visit or later in the follow-up time period. Two thirds of patients (66.0%) did not receive a diagnosis to explain the cause of their FBO (Fig. 2). Remarkably, among the 31 patients (20.7%) who did not undergo endoscopy seven had experienced FBO prior, but only one had a prior diagnosis (oesophageal stricture) that could explain their FBO in 2021. The remaining 30 patients did not have and did not receive a diagnosis to explain the cause of their FBO. None of these 31 patients experienced documented FBO reoccurrence in the study period.

**Table 4:** Pooled diagnoses with relevance to FBO following the initial hospital visit due to FBO and following later hospital visits related to the initial FBO or reoccurrence of FBO

Diagnoses, with relevance to FBO, following the acute hospital visit (including prior known diagnoses), % (n)	
No diagnoses	76.0% (114)
Oesophageal stricture	14.0% (21)
EoE	8.7% (13)
Achalasia	2.0% (3)
Oesophageal or gastric cardia cancer	2.0% (3)
Previous fundoplication surgery	1.3% (2)
Schatzki ring	0.7% (1)
Diagnosis, with relevance to FBO, following later hospital visits related to the initial hospital visit with FBO, or unrelated due to reoccurrence of FBO (including prior known diagnoses), % (n)	
No diagnoses	66.0% (99)
EoE	17.3% (26)
Oesophageal stricture	15.3% (23)
Oesophageal or gastric cardia cancer	5.3% (8)
Schatzki ring	4.0% (6)
Achalasia	2.7% (4)
Previous fundoplication surgery	1.3% (2)

N; number, FBO; food bolus obstruction, EoE; eosinophilic oesophagitis

### Specific ICD-10 codes identified FBO patients

**Fig. 3:**
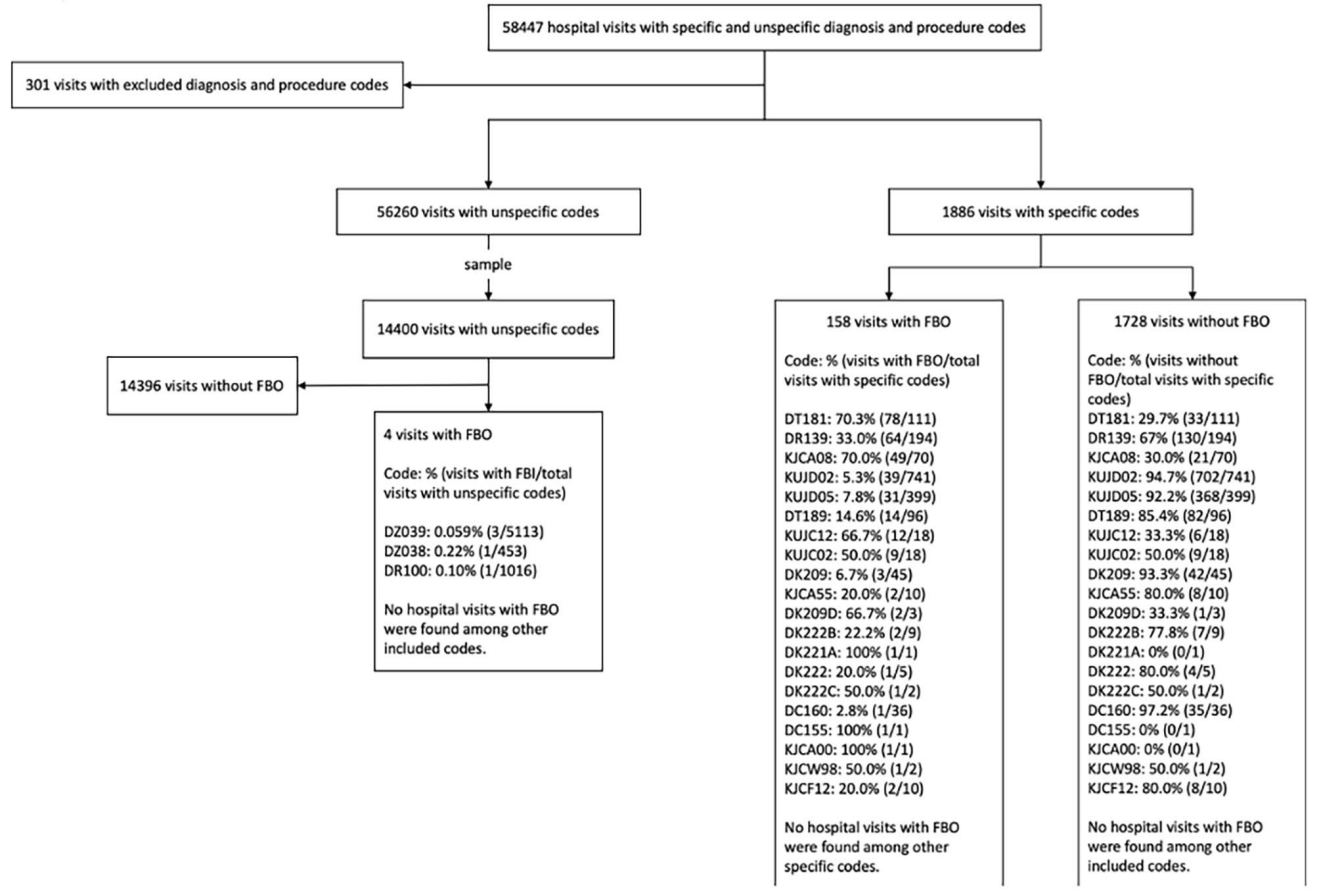
Description of patient sample of visits to departments of Emergency Medicine in NDR in 2021 using ICD-10 and procedure codes. Specific codes were codes possibly covering FBO, and unspecific codes were codes where FBO could not be excluded. Abbreviation: NDR; North Denmark Region, ICD-10; International Classification of Diseases 10th Revision, FBO; food bolus obstruction

Figure 3 shows the filtration of hospital visits with specific and unspecific ICD-10 diagnosis and procedure codes. Hospital visits with unspecific ICD-10 codes (left side of the flow chart), were shown to cover FBO in 0.028% (4 visits among 4 patients) of the sample of 14,400 hospital visits, while hospital visits with specific codes covered FBO in 8.4% (158 visits among 146 patients) of cases (right side of flow chart). Multiple specific ICD-10 codes were often applied to the same FBO visit, while codes DF06, DT180, DC26, KGE, KJN and KJW were not applied to any. Diagnosis codes DT181 and DR139, and procedure code KJCA08 were applied to the greatest number of hospital visits where a patient had oesophageal FBO.

## Discussion

In this retrospective study, we aimed to investigate cases of FBO in the NDR during 2021. Medical records of 150 patients were identified and analyzed, revealing a median age of 66.0 (Q1-Q3: 49.8–81.0) years, with 55.3% having a prior history of FBO. Results showed that 20.7% never underwent upper endoscopy, and 51.3% were never biopsied in the oesophagus. Remarkably, the majority (66.0%) of patients did not receive a definitive diagnosis to explain their FBO symptoms prior to, during, or after their acute hospital visit. ICD-10 codes covering possible FBO showed FBO to be the case in 8.4% of hospital visits. Codes where FBO could not be ruled out covered FBO in 0.028% of hospital visits.

### Patients with FBO were older than comparable studies

The median patient age of 66.0 (Q1-Q3: 49.8–81.0) years is lower than reported by comparable studies [7–11] that also included FBO patients who did not undergo endoscopy. An explanation to this may be that these studies may have included more patients with FBO caused by EoE than this study, which has been reported to occur among younger adults than FBO due to other causes [15]. While this is not directly reflected by the proportions of patients with EoE reported (range: 9.1–22.1%) [7–11], their true proportions are likely all greater than this study (17.3%), since they include patients from and were conducted during time periods where knowledge on EoE was non-existent, limited, or outdated. Supporting this suspicion, the studies report a greater proportion of males ranging from 61.2 to 70.7% [7–11] than this study (58.7%), which closer resemble the male dominant demographic of patients with EoE [16].

### Most patients with FBO never got a diagnosis that can explain why the FBO happened

A majority of patients had no diagnoses with relevance to FBO prior to hospital admission (83.3%), at hospital discharge (76.0%) or following later related hospital visits within the follow-up period (66.0%). These proportions are greater than comparable studies [9, 10] which suggests FBO and its causes need more attention in Denmark. Additionally, 55.3% of patients in this study had a history of previous FBO. Although slightly lower than reported by Fulforth et al. (63.8%) [10], this large proportion indicate that a substantial number of patients may suffer from unidentified and/or insufficiently treated diseases leading to FBO reoccurrence. Delaying the diagnostic work-up in FBO patients can lead to inoperability at diagnosis and death in the case of oesophageal cancer [3]. Delaying oesophageal biopsies can in the case of EoE delay effective treatment with the risk of new FBO [17] which happened to 17.1% of patients in our study. This is greater than the 13.3% reported by Hoversten et al. [9] whose patients were diagnosed with EoE more often and presumable in treatment preventing reoccurrence of FBO.

### Most patients with FBO are not evaluated according to guidelines

A minority of the patients underwent upper endoscopy and histological evaluation according to national guidelines. Specifically, 20.0% (30/150) underwent upper endoscopy within two weeks, and were histologically evaluated within six months following FBO, with at least eight biopsies sampled. The adherence to Danish national guidelines on FBO is difficult to directly compare to other studies, as this is the first Danish study on the topic. Additionally, comparable international studies that report endoscopy and biopsy rates in FBO patients are scarce. However, the upper endoscopy rates of 62.0% within the acute hospital visit and 68.0% within two weeks, are lower than 70.7% reported by Fulforth JM et al. [10] and 82.6% within 1 month by Schupack DA et al. [11], respectively. An explanation to this may be that less patients had endoscopic removal of FBO in this study compared to both Fulforth et al. (62.9%) [10] and Schupack et al. (67.1%) [11]. This might reflect a hesitancy towards performing a diagnostic upper endoscopy among patients that did not require therapeutic upper endoscopy to resolve FBO, as found by Hoversten et al. [9]. The biopsy rates of 24.0% within the acute hospital visit and 45.3% within six months are also unsatisfying as guidelines recommends oesophageal biopsies within two weeks for all patients with FBO. These rates are only slightly higher than those reported by comparable studies within the acute hospital visit (range: 19.0-21.6%) [7, 10] and within 2 years (44.7% from 2007 to 2012) [8] from time periods with lacking knowledge on EoE. This can indicate that EoE only occasionally is suspected as the cause of FBO in the NDR, even though awareness concerning EoE itself has increased in the region [18]. When EoE is not thought of, biopsies may not be sampled as other potentially more known causes of FBO are identified or ruled out without the need of multiple biopsies (e.g., stricture and malignancy). Another explanation for the low biopsy rate within the acute hospital visit may be a possible tendency to postpone biopsy sampling until a later hospital visit as the emergency has been resolved, especially when FBO was not treated with endoscopic removal. This may however lead to a substantial number of patients never being evaluated for EoE as many do not return for outpatient upper endoscopy and biopsy sampling [19]. This and the earlier mentioned hesitancy to refer patients whose FBO resolved without upper endoscopy to outpatient upper endoscopy and biopsy sampling may also explain why only 21.3% of patients had index biopsy sampling from discharge to 6 months after discharge. When biopsies were performed a median of 8.0 (6.0–8.0) were sampled as recommended by Danish national guidelines on FBO [2]. In conclusion more patients with FBO should undergo upper endoscopy and biopsy within two weeks to diagnose esophageal disorders and cancer [20]. Patients with FBO are at risk of having cancer and action should be taken quickly to initiate adequate treatment.

### Unspecific ICD-10 codes were almost never applied to hospital visits due to FBO

Almost no hospital visits with only unspecific ICD-10 codes applied were due to FBO (0.028%). With an assumption of similar ICD-10 code appliance tendencies in populations outside the NDR, this finding enables future studies to exclude hospital visits with only unspecific ICD-10 codes applied with minimal loss in search of hospital visits due to FBO. This rate was expectedly lower than 8.4% hospital visits due to FBO among hospital visits with specific ICD-10 and procedure codes applied. No single code managed to identify a majority of hospital visits due to FBO with specific codes applied (DT181 identified 49.4%). This is most likely because no ICD-10 code specifically for FBO exists. However, the combination of codes DR139, DT181, DT189 and KJCA could possibly be used as a satisfying proxy as it identifies 95.2% of the 146 patients that had a hospital visit due to FBO with specific codes applied in this study. This while lowering the number of hospital visits to review from 1886 to 393. As this combination of codes still captures 245 irrelevant hospital visits, a manual screening of the 393 hospital visits for FBO episodes would still be necessary.

### Strengths and limitations

A strength of this study is that all ICD-10 and procedure codes assumed possible to be applied to hospital visits due to FBO were included, as well as codes where FBO could not be excluded. As such, it is very likely that almost if not all hospital visits due to FBO in the NDR in 2021 are included in this study. Despite this, an important limitation is that there is no ICD-10 code specific for FBO, and as such, the identification of hospital visits due to FBO depend greatly on the selection of codes deemed possible to be applied to these. Another limitation is that the reoccurrence rate found in this study only include occurrences documented in the medical files of the patients. As such, FBO solved at home without notifying the hospital or necessitating a hospital visit are not included, and the actual proportion that experienced FBO reoccurrence may be even larger.

## Conclusion

Most hospitalized FBO patients in the NDR in 2021 were never diagnosed with a cause. In these patients there is a high risk of overlooked upper gastrointestinal cancers or EoE. The area needs immediate focus and changed routines to improve treatment and prevent new FBO.

## Data Availability

The datasets generated and/or analyzed during the current study are not publicly available due to patient sensitive information but are available from the corresponding author on reasonable request.

## Abbreviations

FBO: Food bolus obstruction
EoE: Eosinophilic oesophagitis
NDR: North Denmark Region
SD: Standard deviation
ICD-10: International Classification of Diseases 10th Revision
IQR: Interquartile range
